# Chronic treatment with anesthetic propofol attenuates β-amyloid protein levels in brain tissues of aged mice

**DOI:** 10.1186/2047-9158-3-8

**Published:** 2014-04-11

**Authors:** Yiying Zhang, Haijun Shao, Yuanlin Dong, Celeste A Swain, Buwei Yu, Weiming Xia, Zhongcong Xie

**Affiliations:** 1 Geriatric Anesthesia Research Unit, Department of Anesthesia, Critical Care and Pain Medicine, Massachusetts General Hospital and Harvard Medical School, 149 13th St., Room 4310, Charlestown, MA 02129-2060, USA; 2 Department of Anesthesiology, Ruijin Hospital, Shanghai Jiao Tong University School of Medicine, 200025 Shanghai, P.R. China; 3 Department of Veterans Affairs, Medical Research and Development Service and Geriatric Research, Education and Clinical Center, Bedford, MA 01730, UK

**Keywords:** Aging, Alzheimer’s disease, Neurodegeneration, β-amyloid peptide, Anesthesia, Propofol

## Abstract

Alzheimer’s disease (AD) is the most common form of dementia. At the present time, however, AD still lacks effective treatments. Our recent studies showed that chronic treatment with anesthetic propofol attenuated brain caspase-3 activation and improved cognitive function in aged mice. Accumulation of β-amyloid protein (Aβ) is a major component of the neuropathogenesis of AD dementia and cognitive impairment. We therefore set out to determine the effects of chronic treatment with propofol on Aβ levels in brain tissues of aged mice. Propofol (50 mg/kg) was administrated to aged (18 month-old) wild-type mice once a week for 8 weeks. The brain tissues of mice were harvested one day after the final propofol treatment. The harvested brain tissues were then subjected to enzyme-linked immunosorbent assay (ELISA) and Western blot analysis. Here we report that the propofol treatment reduced Aβ (Aβ40 and Aβ42) levels in the brain tissues of the aged mice. Moreover, the propofol treatment decreased the levels of β-site amyloid precursor protein cleaving enzyme (the enzyme for Aβ generation), and increased the levels of neprilysin (the enzyme for Aβ degradation) in the brain tissues of the aged mice. These results suggested that the chronic treatment with propofol might reduce brain Aβ levels potentially via decreasing brain levels of β-site amyloid precursor protein cleaving enzyme, thus decreasing Aβ generation; and via increasing brain neprilysin levels, thus increasing Aβ degradation. These preliminary findings from our pilot studies have established a system and postulated a new hypothesis for future research.

## Introduction

Alzheimer disease (AD) is an insidious and progressive neurodegenerative disorder accounting for the vast majority of dementia, and is characterized by global cognitive decline and the robust accumulation of amyloid deposits and neurofibrillary tangles in the brain (reviewed in [1]). However, there is still a lack of effective treatments for AD, and many studies aim to find new and novel drugs to treat and/or prevent AD.

β-Amyloid protein (Aβ) is a main component of the plaques found in brains of AD patients and is the hallmark of AD dementia and cognitive impairment (reviewed in [1]). Accumulation and deposition of β-amyloid protein (Aβ) has been reported as one of the main aspects of AD neuropathogenesis ([2-4], reviewed in [1]). Aβ was isolated from meningovascular amyloid deposits in AD and Down’s syndrome [2,5]. These findings led to the cloning of the gene encoding amyloid precursor protein (APP) as one of the AD genes [6,7] and consequently, the further studies of APP processing and Aβ metabolism.

APP is hydrolyzed by aspartyl protease β-site APP-cleaving enzyme (BACE) or β-secretase, a type I transmembrane, glycosylated aspartyl protease found in post-Golgi membranes and at the cell surface [8-11], and then is cleaved by γ-secretase [12-14] to generate Aβ. Finally, Aβ can be degraded by enzyme insulin degrading enzyme (IDE) and neprilysin (NEP) ([15-19]; reviewed in [20]).

Propofol (2, 6-disopropylphenol) is an intravenous anesthetic. It has been shown that propofol can attenuate the caspase-3 activation and Aβ oligomerization induced by the anesthetic isoflurane [21]. Our recent studies have shown that chronic treatment with propofol (50 mg/kg, once per week for 8 weeks) in aged mice (e.g., 18 month-old) can improve the cognitive function and attenuate the caspase-3 activation [22]. Given Aβ accumulation can lead to cognitive impairment [reviewed in [1]), we set out in the present pilot studies to establish a system and to test a hypothesis that the chronic treatment with propofol can decrease Aβ levels in the brain tissues of aged mice via inhibiting its generation and/or promoting its degradation. The findings from these proof of concept studies would promote more research to further determine the effects of anesthetic propofol on AD neuropathogenesis.

## Materials and methods

### Mice and propofol treatment

The animal protocol was approved by the Standing Committee on Animals at Massachusetts General Hospital, Boston, Massachusetts. The maintenance and handling of mice were consistent with the guideline of National Institute of Health, and all of the efforts were made to minimize the number of animals in the studies. Wild-type mice (C57BL/6 J, The Jackson Lab, Bar Harbor, ME) were used in the study. There were 10 mice in the propofol treatment group and 10 mice in the saline control group. The mice, at the age of 18 months-old, were randomized by weight and gender into experimental groups, which received propofol (APP Pharmaceuticals, Inc, Schaumburg, IL) treatment [50 mg/kg, intraperitoneal (IP) injection], and control groups, which received the same volume of saline (IP), once a week every Saturday for 8 weeks.

### Tissue preparation

One day after the last propofol or saline treatment, mice were decapitated, and the brain tissues were harvested. The harvested brain tissues were homogenized on ice with an immunoprecipitation buffer (10 mM Tris–HCl, pH 7.4, 150 mM NaCl, 2 mM ethylenediaminetetraacetic acid, 0.5% Nonidet P-40) plus protease inhibitors (1 μg/ml aprotinin, 1 μg/ml leupeptin, 1 μg/ml pepstatin A). The lysates were centrifuged at 14,000 rpm for 15 minutes, and quantified for total protein concentration by a bicinchoninic acid protein assay kit (Pierce, Iselin, NJ). The harvested brain tissues were subjected to Enzyme-linked immunosorbent assay (ELISA) or Western blot analyses as described in our previous studies [21,23,24].

### ELISA determination of A β

The mouse Aβ40 (KMB3481) and Aβ42 (KMB3441) immunoassay Kits (Invitrogen, San Francisco, CA) were used to determine the Aβ40 and Aβ42 levels in the brain tissues of the aged mice, respectively. The brain tissues were homogenized in TBS buffer (including 50 mM Tris, and 150 mM NaCl, pH 8.0) with protease inhibitor (1 mg/ml aprotinin, 1 mg/ml leupeptin, 1 mg/ml pepstatin A), and then centrifuged for 45 minutes at 65,000 rounds per minute (RPM) at 4 degrees Celsius. The supernatants were removed. The pellets were resuspended by sonication in a homogenization buffer containing 1% SDS, and spun again (15 minutes at 18,000 RPM). We collected the supernatants and measured the total protein amount of each sample. We obtained 110 μg of protein from each harvested mouse brain sample or standard, and placed the sample into each well coated with a monoclonal antibody to the NH2-terminus of mouse Aβ. The samples and the antibody were incubated overnight at 4 degrees Celsius. After washing 4 times, a rabbit monoclonal antibody specific for the COOH-terminus of the Aβ sequence (1–40 or 1–42) was added and incubated in room temperature for one hour. After another 4 washes, horseradish peroxidase-labeled anti-rabbit antibody was added to the wells, and incubated for a half hour at room temperature. Wells were then developed with tetramethylbenzidine (TMB) reagent in dark and the well absorbance was measured at 450 nm. Aβ40 and Aβ42 levels in test samples were determined by comparison with the signals from the standard spiked with known quantities of Aβ40 or Aβ42.

### Western blot analysis

BACE antibody (1:1,000 dilution; Abcam, Cambridge, MA, Cat. Number: ab2077) was used to recognize BACE (65 kDa). Anti-neprilysin (NEP) antibody (1:1,000 dilution, Millipore, Temecula, CA) was used to detect protein levels of NEP (86 kDa). Antibody anti-β-Actin (1:10,000, Sigma, St. Louis, MO) was used to detect β-Actin (42 kDa). Each band in the Western blot represented an independent experiment. The results were averaged from 6–10 independent experiments. We quantified the Western blots in two steps as described in our previous studies [25]. First, we used β-Actin levels to normalize protein levels (e.g., determining the ratio of BACE to β-Actin amount) and control for loading differences in the total protein amount. Second, we presented protein level changes in the brain tissues of mice treated with propofol as a percentage of those in the saline control group. 100% of protein level changes refer to control levels for the purpose of comparison to experimental conditions.

### Immunoblot detection of Aβ

Immunoblot detection of Aβ in brain tissues was measured as described in previous studies [25-27]. Specifically, brain samples were homogenized (150 mM NaCl with protease inhibitor cocktail in 50 mM Tris, pH of 8.0) and centrifuged (65,000 rpm × 45 minutes), and the supernatant was removed. The pellet was then resuspended by sonication in homogenization buffer containing 1% SDS. Following the pelleting of insoluble material (18,000 rpm × 15 minutes), the SDS-extract was electrophoresed on SDS-PAGE (4-12% Bis-Tris polyacrylamide gel from Invitrogen, Carlsbad, CA), blotted to PVDF membrane and probed with a 1:200 dilution of 6E10 (Covance).

### Statistics

Data were expressed as mean ± standard deviation (SD). The number of samples varied from 6 to 10, and the samples were normally distributed (tested by normality test). Student-t test was used to analyze the difference in Aβ, BACE, and NEP levels between the brain tissues of the propofol-treated mice and the brain tissues of the saline-treated mice. Prism 6 software (La Jolla, CA) was used to analyze the data.

## Results

### Propofol treatment reduced the Aβ levels in the brain tissues of aged mice

Our recent studies [28] showed that chronic treatment with anesthetic propofol (50 mg/kg, once a week for 8 weeks) was able to improve cognitive function and attenuated the aging-associated caspase-3 activation. Both caspase-3 activation and Aβ have been reported to contribute to AD neuropathogenesis and cognitive impairment ([29], reviewed in [1]). Therefore, we set out to determine whether the chronic propofol treatment could also reduce the Aβ levels in the brain tissues of mice.

**Figure 1:**
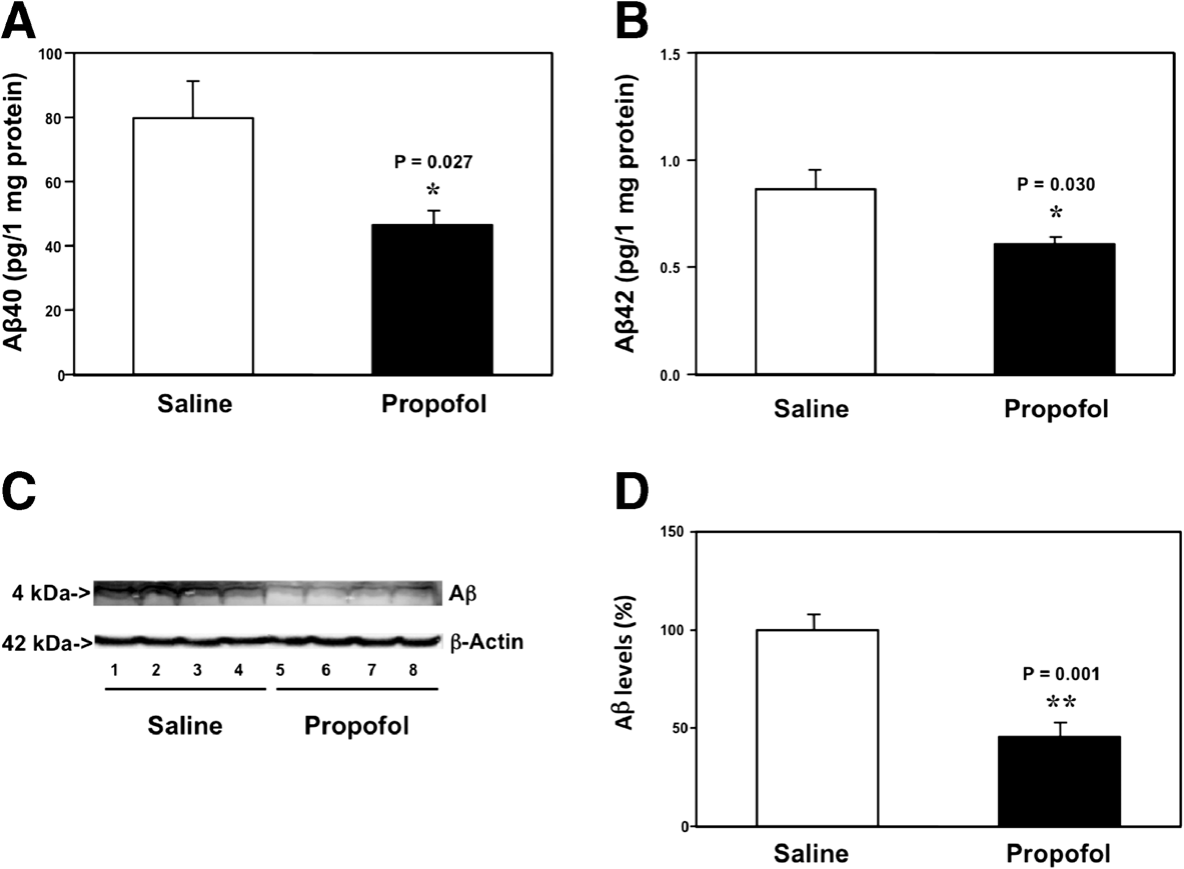
**Propofol decreases Aβ levels in the brain tissues of aged mice. A**. ELISA shows that there are lower levels of Aβ40 in the brain tissues of mice following the propofol treatment (black bar) as compared to the mice following the saline treatment (white bar). **B**. ELISA shows that there are lower levels of Aβ42 in the brain tissues of mice following the propofol treatment (black bar) as compared to the mice following saline treatment (white bar). **C**. Western blot analysis shows that there are lower levels of Aβ in the brain tissues of mice following propofol treatment (lanes 5 to 8) as compared to the mice following saline treatment (lanes 1 to 4). **D**. Quantification of the Western blot shows that there are lower levels of Aβ in the brain tissues of mice following propofol treatment (black bar) as compared to the mice following saline treatment (white bar). N = 10.

The 18 month-old mice received 50 mg/kg propofol or saline once a week for 8 weeks. On the day after the last propofol treatment, the mice were euthanized and the brain tissues were harvested. The harvested brain tissues were subjected to ELISA studies for the determination of Aβ40 and Aβ42 levels. The ELISA studies showed that the brain tissues from the propofol-treated mice had lower levels of Aβ40 as compared to the brain tissues from the saline-treated mice: 42 versus 78 pg/ 1 mg protein, P = 0.027 (Figure 1A). The ELISA studies also showed that the propofol treatment reduced the Aβ42 levels in the brain tissues of mice: 0.54 versus 0.89 pg/ 1 mg protein, P = 0.030 (Figure 1B). These data suggested that the chronic treatment with propofol might decrease both Aβ40 and Aβ42 levels in brain tissues of aged mice.

Next, we performed Western blot analysis of Aβ to further determine the effects of the propofol treatment on the Aβ levels in the brain tissues of the aged mice. The immunoblotting of Aβ showed that there was a visible reduction in the levels of bands representing Aβ (4 kDa) in the brain tissues of mice following the propofol treatment as compared to those of mice following the saline treatment (Figure 1C). There was no significant difference in the β-Actin levels between the brain tissues of mice following the propofol treatment and the brain tissues of mice following the saline treatment. The quantification of the Western blot, based on the ratio of Aβ to β-Actin, showed that the propofol treatment (black bar) decreased the Aβ levels as compared to the saline treatment (white bar): 43% versus 100%, P = 0.001 (Figure 1D). Taken together, these data suggested that the chronic treatment with 50 mg/kg propofol weekly for 8 weeks was able to decrease Aβ levels in the brain tissues of aged mice.

### Propofol treatment reduced the BACE levels in the brain tissues of aged mice

**Figure 2:**
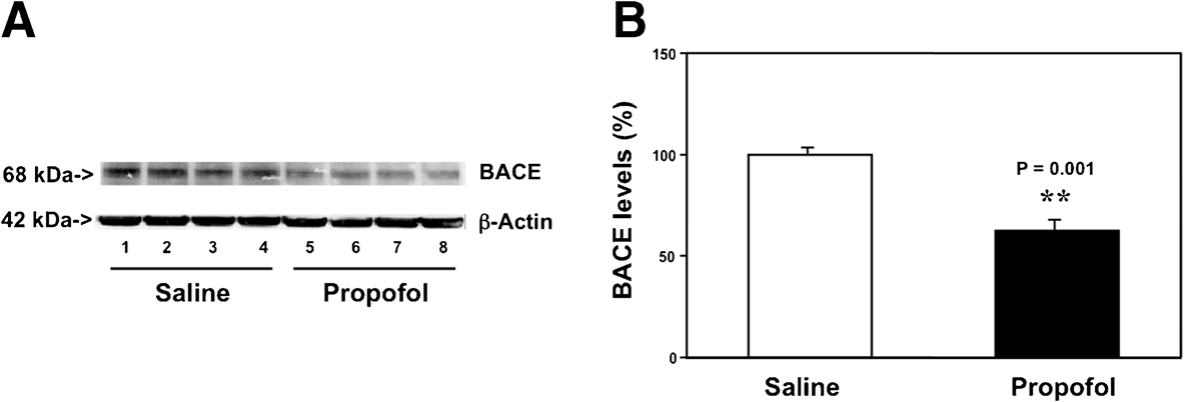
**Propofol decreases BACE levels in the brain tissues of aged mice. A**. Western blot analysis shows that there are lower levels of BACE in the brain tissues of mice following propofol treatment (lanes 5 to 8) as compared to the mice following saline treatment (lanes 1 to 4). **B**. Quantification of the Western blot shows that there are lower levels of BACE in the brain tissues of mice following propofol treatment (black bar) as compared to the mice following saline treatment (white bar). N = 6.

Given the findings that the propofol treatment could decrease the Aβ levels in the brain tissues of aged mice, next, we investigated the potential underlying mechanisms. We used Western blot analysis to assess the effects of the propofol treatment on the level of BACE, the enzyme for the Aβ generation [8]. The BACE immunoblotting showed that there were reduced levels in the levels of the bands representing BACE (65 kDa) in the brain tissues of the propofol-treated mice (lanes 5 to 8) as compared to those of the saline-treated mice (lanes 1 to 4) (Figure 2A). The quantification of the Western blot showed that the propofol treatment (black bar) decreased the BACE levels as compared to saline treatment (white bar): 58% versus 100%, P = 0.001 (Figure 2B). These results suggested that the propofol treatment might decrease Aβ levels by reducing its generation through inhibition of its generation enzyme, BACE.

### Propofol treatment increased the NEP levels in the brain tissues of aged mice

**Figure 3:**
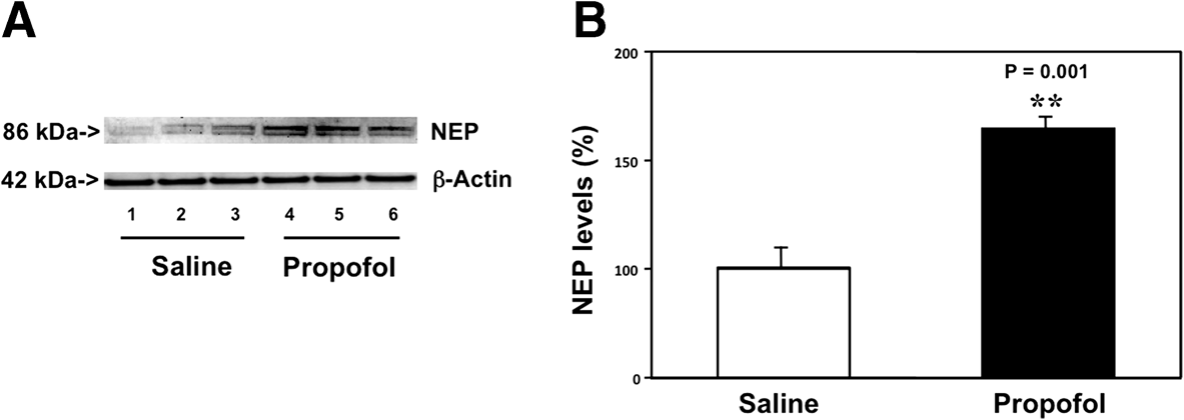
**Propofol increases NEP levels in the brain tissues of aged mice. A**. Western blot analysis shows that there are higher levels of NEP in the brain tissues of mice following propofol treatment (lanes 4 to 6) as compared to the mice following saline treatment (lanes 1 to 3). **B**. Quantification of the Western blot shows that there are higher levels of NEP in the brain tissues of mice following propofol treatment (black bar) as compared to the mice following saline treatment (white bar). N = 6.

The reduction of Aβ could be due to either a decrease in its generation (e.g., decrease in BACE levels) or increase in its degradation. Neprilysin (NEP) is one of the enzymes of Aβ degradation ([15-19]; reviewed in [20]). We therefore assessed the effects of the chronic propofol treatment on the levels of NEP in the brain tissues of the aged mice by employing the Western blot analysis. The NEP immunoblotting showed visible increases in the levels of bands representing NEP (86 kDa) in the mice treated with propofol (lanes 4 to 6) as compared to those treated with saline (lanes 1 to 3) (Figure 3A). The quantification of the Western blot showed that the propofol treatment (black bar) increased the levels of NEP as compared to the saline treatment (white bar) in the brain tissues of the aged mice: 167% versus 100%, P = 0.001 (Figure 3B). These data suggested that the propofol treatment might also decrease Aβ levels by increasing its degradation through promotion of its degradation enzyme, NEP.

## Discussion

Our recent studies have shown that a weekly treatment with 50 mg/kg propofol for 8 weeks is able to improve the cognitive function in the aged mice, and reduces caspase-3 activation in the brain tissues of the mice [22]. Aβ accumulation also contributes to cognitive impairment [reviewed in [1]). Therefore, in the current study, we assessed the effects of the chronic treatment of propofol on the levels of Aβ, as well as its generation enzyme BACE and degradation enzyme NEP. Note that the main objective in this proof of concept study is to establish a pre-clinical model for future large scale studies.

We found that the weekly treatment of propofol for 8 weeks in the aged mice was able to attenuate the Aβ levels in the brain tissues of the mice (Figure 1). These data suggested that it was possible that the anesthetic propofol might improve cognitive function in aged mice by reducing the Aβ levels in the brain tissues of the mice. However, the cause-effect relationship of the propofol-induced reduction in brain Aβ levels of aged mice and the propofol-induced improvement of cognitive function in aged mice remain to be determined. Such studies would illustrate the functional outcomes of the propofol-induced reduction in brain Aβ levels of aged mice and are warranted to perform in the future.

Moreover, the propofol treatment decreased BACE levels in the brain tissues of the aged mice (Figure 2). These results showed the potential underlying mechanism of the propofol-induced reduction in Aβ levels, and suggested that propofol might decrease the Aβ levels by inhibiting its generation in the brain tissues of the mice.

Finally, the propofol treatment increased the NEP levels in the brain tissues of the aged mice (Figure 3). These findings demonstrated a different underlying mechanism of the propofol-induced reduction in Aβ levels, and suggested that propofol might decrease the Aβ levels by enhancing its degradation in the brain tissues of the mice.

A recent study has shown that AD patients may have an age-dependent decrease of gamma-aminobutyric acid (GABA) currents in the AD brain, and this reduction is associated with decreased mRNA and protein levels of GABA receptor subunits [30]. These findings suggest that reduced GABA neurotransmission could also contribute to AD neuropathogenesis. Propofol is a GABA receptor agonist [31], and it has been shown in a preliminary clinical study that propofol may improve cognitive function in humans [32]. Our current studies showed that the chronic propofol treatment might decrease BACE levels (the generation enzyme) and increase NEP levels (the degradation enzyme), leading to reduction in Aβ levels in brain tissues of aged mice. Furthermore, our recently published work [22] suggested that the same chronic propofol treatment was able to attenuate caspase-3 activation in the brain tissues of aged mice and improved cognitive function in the mice. Taken together, these findings suggest the potential association between GABA neurotransmission with caspase activation, Aβ metabolism and cognitive function. Future studies may use different GABA receptor agonist(s) to further test this hypothesis. These findings may promote more research, leading to new concepts of AD neuropathogenesis and new intervention(s) of AD.

Moreover, these findings demonstrated the possibility that the anesthetic propofol could be used to prevent or treat neurological disorders, e.g., AD. Pending further studies, the chronic treatment with propofol would be used to attenuate the neuropathogenesis of AD and to improve the cognitive function in AD patients. These studies would promote further investigations, in both pre-clinical and clinical settings, to seek innovative uses of current anesthetics for the interventions of other disorders.

Some anesthetics, e.g., isoflurane, have been shown to induce neurotoxicity and neurobehavioral deficits *in vitro* and *in vivo*[25,33-46]. Therefore, the current findings that propofol attenuated Aβ levels in brain tissues of mice suggested that more studies are needed to assess whether propofol could be a better choice when providing anesthesia care for AD patients or senior patients who are vulnerable to develop postoperative cognitive dysfunction.

Note that propofol is a short acting anesthetic agent. The observed reductions in the levels of Aβ and BACE, and increases in the NEP levels were likely not the acute effects of propofol. The exactly mechanism by which the chronic treatment of propofol alters the levels of Aβ, BACE, and NEP remains unknown at the current time. We have postulated that the weekly treatment with 50 mg/kg propofol for 8 weeks may regulate the functional status of GABA receptor, which then leads to the changes in the levels of Aβ, BACE, and NEP. Future studies to test this hypothesis are warranted.

The studies have several limitations. First, we did not determine the dose or time-dependent effects of propofol on Aβ levels in the brain tissues of the aged mice. Different treatments of propofol may be neurotoxic [47-49] or neuroprotective [50-52]. Therefore, it is possible that propofol treatment with different doses or administered at different times may have different effects on brain Aβ levels. Nevertheless, the outcomes from the current studies have established a system and proposed a new concept to further determine the effects of propofol on brain function. Second, we did not assess the effects of propofol on the levels of other enzymes involving in Aβ metabolism, e.g., γ-secretase and/or insulin degradation enzyme (IDE). However, the main objective of the current studies was to determine whether anesthetic propofol could decrease brain Aβ levels in aged mice. We will systematically investigate the underlying mechanism by which propofol affects brain Aβ levels using our established system in the future.

In conclusion, we found that chronic treatment with the anesthetic propofol was able to reduce Aβ (both Aβ40 and Aβ42) levels in the brain tissues of aged mice. Furthermore, the chronic propofol treatment might reduce the brain Aβ levels by decreasing brain BACE levels (decreasing Aβ generation) and increasing brain NEP levels (increasing Aβ degradation). The findings from these concept and hypothesis generation studies will promote more research to systematically determine the effect of chronic treatment of propofol or other anesthetics on Aβ levels and the associated behavioral changes, which would ultimately lead to the development of new therapeutic strategies for aging- and/or AD-associated cognitive impairment and of better anesthesia care for senior and AD patients.

## Abbreviations

AD: Alzheimer’s disease; APP: Amyloid β precursor protein; BACE: β-site amyloid precursor protein cleaving enzyme; NEP: Neprilysin.

## Competing interests

The authors have no conflicts of interest for the study.

## Authors’ contributions

YZ and ZX designed the experiments; YZ, HS and YD carried our the experiments; YZ, CS and BY analyzed the data; WX and ZX wrote the paper. All authors read and approved the final manuscript.
